# Basic motor competencies in Slovak children from the 3rd and 4th grade elementary age group

**DOI:** 10.3389/fped.2024.1175468

**Published:** 2024-01-29

**Authors:** Ľuboslav Šiška, Peter Mačura, Andrej Hubinák, Peter Krška, Jaromír Sedláček, Anna Blahutová, Martin Zvonař, Katarína Kohútová, Lovro Štefan

**Affiliations:** ^1^ Department of Physical Education and Sports, Faculty of Education, Catholic University in Ružomberok, Ružomberok, Slovak Republic; ^2^ Department of Sport Kinantropology, Faculty of Sports, Prešov University in Prešov, Prešov, Slovak Republic; ^3^ Division of Sport Motorics and Methodology in Kinanthropology, Faculty of Sports Studies, Masaryk University in Brno, Brno, Czech Republic; ^4^ Department of Social Work, Faculty of Education, Catholic University in Ružomberok, Ružomberok, Slovak Republic; ^5^ Department of General and Applied Kinesiology, Faculty of Kinesiology, University of Zagreb, Zagreb, Croatia; ^6^ Department of Physical Activities and Health Sciences, Faculty of Sports Studies, Masaryk University, Brno, Czech Republic; ^7^ Department of Recruitment and Examination (RECETOX), Faculty of Science, Masaryk University, Brno, Czech Republic

**Keywords:** basic motor competence, third and fourth graders, elementary schools, boys and girls, MOBAK 3−4 test battery

## Abstract

**Background:**

The development of movement skills is the basic goal of physical and sports education. Their level is a determining factor in the subsequent involvement of the child in physical activities. Goal is to contribute to the knowledge of basic motor competencies (BMC) in the 3rd and 4th grade boys and girls from elementary schools.

**Methods:**

We collected data from 468 children (Mage = 9.6, SD = 0.6 years; 228 girls and 240 boys) from 16 third grade and 16 fourth grade classes at ten elementary schools in different parts (central, west, north) of the Slovak Republic. The primary data on the basic motor competencies of the examined groups were obtained by means of the MOBAK-3 test battery. Construct validity was analyzed by confirmatory factorial analysis and relationships between BMC and age, gender and BMI were analyzed by structural equation modeling.

**Results:**

Boys had a better performance in object movement activities than the girls *p* < 0.01. Situation in self-movement activities was different, girls had a better performance than boys *p* < 0.05. The general expectation that grade is a factor to improve the performance in basic motor competencies in the compared 3rd- and 4th-graders was not clearly fulfilled. This assumption was only confirmed in girls (object movement *p* < 0.05, self movement *p* < 0.01). Confirmatory factor analysis confirmed the two-factor structure of basic motor competencies on object-movement and self-movement, while gender, BMI and age were confirmed as significant covariances.

**Conclusions:**

The theory of BMC in the 3rd- and 4th-graders at elementary schools is an appropriate method to uncover the grounds for a positive attitude to physical activities later in life.

## 1 Introduction

In recent decades, researches on motor competence have taken an important place for the needs of education. BMC are becoming one of the most important topics in physical education (1, 2). Physical education in primary school enables children to acquire and develop basic motor competences that strengthen the child's healthy motor development and encourages participation in physical and sports activities with peers (3, 4). The level of BMC affects not only the child's physical development, but also cognition, mental and social aspects (2, 5–7), while clarifying the importance of motor competences should be a vision of the holistic development of children at school (2, 8, 9). The stated targets are elaborated in European (European Commission/EACEA/Eurydice 2013) and non-European (e.g., SHAPE America 2014) mandatory pedagogical documents for teachers of physical education, as the subject of physical education is generally a compulsory subject in primary schools (4).

Motor competence is a latent functional performance disposition, which can be observed through fundamental motor skills and divided into two categories: one linked to self-movement (or locomotion), which is the movement and control of the body in an open space (e.g., running, jumping, rolling) and one related to skills that make it possible to control objects, which involve using the hands and feet to manipulate them or project them (throwing, catching, dribbling) (10). Learning outcomes are obtained by evaluating pupils (11), which also concerns physical education. Teachers detect the child's lagging behind in basic motor competencies, learning outcomes and improve their teaching procedures (12).

Therefore, the evaluation tool MOBAK (abbreviation of the German words Motorische Basiskompetenzen) was created with the aim of determining the level of motor competence in order to improve the school curriculum of education firstly aimed at children in primary education (13). Following the product- and process-oriented evaluation of competencies in children to perform physical activities (14), the test batteries can be divided in a similar way. The set of four MOBAK test batteries (1, 15–17) belongs to the former, and Eurofit (18) to the latter category. The MOBAK test batteries have been used increasingly in the recent years to identify the BMC in children from preschool to the beginning of the second grade of primary school in many European countries (1, 13), but also in other countries (2). The basic (preliminary) findings show that boys outperform girls in object movement tasks and, on the other hand, girls tend to show slightly better results in self-movement tasks (2, 4, 10, 12). In the Slovak Republic, we only collected the data in Southern Slovakia (4); the remaining regions have not been analyzed yet. It was also necessary to confirm the construct validity when dividing the competencies into object movement and self-movement (1, 2, 10, 12, 13), and this issue has not been addressed in Slovakia at all.

Multiple individual factors are associated with BMC. Endogenous factors such as age, sex and body mass index (BMI) have been assessed consistently in basic motor competency research. In Carcamo research (2) all of the factors were significant but in other research (1, 4, 15) results were not so clear.

Realizing the importance of assessing the level of BMC in physical education (2), the main aim of the paper is to contribute to the knowledge of basic motor competencies in the 3rd and 4th grade boys and girls from elementary schools in the Slovak Republic and to determine the construct validity of the MOBAK battery also with the covariates gender, BMI and age.

## 2 Materials and methods

### 2.1 Participants

In our research we used non-probability convenience sampling method based on non-random criteria and we collected data from 468 boys and girls from 16 third grade and 16 fourth grade classes at ten elementary schools in different part (Central, West, North) of the Slovak Republic (Table 1).

**Table 1:** Descriptive parameters of compared groups.

Grade	Gender	*n*	Age (years)		Body weight (kg)		Body height (cm)		BMI	
			M	SD	M	SD	M	SD	M	SD
3rd	boys	116	9.24	0.42	36.10	10.74	139.92	6.64	18.22	4.16
	girls	116	9.08	0.41	34.77	8.07	137.33	6.50	18.29	3.27
	total	232	9.16	0.42	35.43	9.50	138.62	6.69	18.25	3.73
4th	boys	124	10.03	0.38	39.45	9.61	143.79	6.97	18.96	3.80
	girls	112	10.04	0.41	35.29	7.23	141.92	7.06	17.41	2.64
	total	236	10.03	0.39	37.48	8.79	142.91	7.06	18.22	3.38
3rd + 4th	boys	240	9.65	0.56	37.83	10.29	141.92	7.07	18.60	3.99
	girls	228	9.55	0.63	35.03	7.65	139.59	7.14	17.86	3.00
	total	468	9.60	0.60	36.46	9.20	140.78	7.19	18.24	3.56

491 children started MOBAK test battery. For pedagogical reasons, we tested all of them. Upon determining that a particular child is incapable of perform-ing the test task for health and safety reasons, we disallowed him/her to perform the test task. The processing of raw data only included those children that did not show any obvious health-threatening and other motor dysfunctions, and/or health ailment (e.g., missing arm etc.), and we fully followed the Declaration of Helsinki.

### 2.2 Instruments

The MOBAK-3 battery is structured around eight motor tasks (observable items) that cover the object movement and self-movement motor competencies (latent factors). The object movement competence includes the motor tasks:

Throwing—The student throws six juggling balls from a 3 m distance at a target.

Catching—The student throws up a ball and catches it behind a line at a 1.5 m distance.

Bouncing a ball—The student bounces a small basketball back and forth through a marked corridor (7.5 × 1.4 m) with obstacles without losing the ball.

Dribbling—The student dribbles a football back and forth through a marked corridor (7.5 × 1.4 m) with obstacles without losing the ball.

The self-movement competence includes the motor tasks:

Balancing—The student balances back and forth across a long upside-down bench with two boxes attached that have to be overstepped. No follow steps.

Rolling—The student performs a fluent forward roll, starting with a jump onto a pair of vaulting boxes.

Jumping—The student skips continuously on the spot for 20 s.

Running—The student follows the markings, forming an eight by moving forward or sideways around the cones.

The children have two attempts at each item, apart from throwing and catching where they have six attempts. The tests are marked on a dichotomous scale (0 = failed; 1 = successful), recording the number of successful attempts (no successful attempts = 0 points; one successful attempt = 1 point; two successful attempts = 2 points). For the throwing and catching tasks, the children have six attempts, with the number of successful attempts being scored as follows: 0–2 successful attempts = 0 points; 3–4 successful attempts = 1 point; and 5–6 successful attempts = 2 points (2, 10).

### 2.3 Procedure

14 schools were addressed, and the research was attended by 5 schools located in cities and 5 schools located in rural areas. In January 2019, we conducted a two-day pilot testing of one 3rd grade class (*n* = 18) and one 4th grade class (*n* = 19) at an elementary school in order to gain some experience with the MOBAK 3–4 test battery. The pilot testing was preceded by a detailed briefing for the administrators in the gym. The administrators were provided the Test manual (19), the publication (20) and the handbook (21). The testing was performed with the approval of regional education management institutions, school management, parents and children. Participation was voluntary. The data were collected during standard classes in the school gym. The testing took place from January 2019 to November 2019.

The testing was conducted by previously instructed and practically trained administrators—university teachers of physical education and sports and students—future teachers at the preschool and junior elementary level. The children were tested with the test battery for the first time (in their lives) and as per the administrating instructions. The test was conducted by the test administrators with entire school classes and during regular school hours, like Scheuer, Bund & Herrmann (22). The Ethics Committee at the Faculty of Education, Catholic University in Ružomberok, Slovak Republic, confirmed the compliance with the rules of ethics in human research. All subjects gave their informed consents for inclusion before they participated in the study.

### 2.4 Data analysis

The statistical analyses were done in MS Excel 2016, IBM SPSS 22 with significance levels of *p* < .05 and *p* < .01. Collected data were analyzed by means of descriptive statistic, means (M) and standard deviations (SD). The significance of the difference between the two compared groups was calculated and evaluated by Student's *t*-test in two independent groups. With JASP 0.16.4.0 software we calculated a confirmatory factor analytic model to test for construct validity. In Model 1, we tested the MOBAK 1-test instrument to confirm its factorial structure. The four test items balancing, rolling, jumping, and running were assigned to the factor self-movement, and the four test items throwing, catching, bouncing, and dribbling were assigned to the factor object movement (10, 13). Secondary loadings of test items on the no assigned factor were not allowed. Factor loadings and residual variances were estimated freely for each test item. On the basis of the two-factorial solution (Model 1), we calculated the Model 2 with the covariates BMI, age and gender to identify the determinants of basic motor competencies (12). In both models, the items from the MOBAK battery were treated as an ordinal scale and the ML (Maximum Likelihood) estimator was used with the MPLUS emulation and robust error calculation. To assess the fit of the models the RMSEA (Root Mean Square Error of Approximation) and CFI (Comparative Fit Index) indices were used, with values lower than.06 for RMSEA or over.90 for CFI being regarded as acceptable (2, 23).

## 3 Results

When evaluating individual motor competencies, we can observe that fourth-graders achieve better performance in tasks related to object control (throwing, catching, bouncing, dribbling) than third-graders, but not statistically significant *p* > 0.05. However, the level is significantly higher for parameters related to body movement: balancing t(466) = −1.99, *p* < .05, rolling t(466) = −3.06, *p* < .01, jumping t(466) = −2.89, *p* < .01 and running t(466) = −3.90, *p* < .01. If we compare the level of movement competences between boys and girls in the third and fourth grades together in object movement, boys significantly dominate over girls in throwing t(466) = 3.08, *p* < .01, catching t(466) = 4.32, *p* < .01, bouncing t(466) = 4.75, *p* < .01 and dribbling t(466) = 7.58, *p* < .01. When it comes to self-movement, boys have a higher level in rolling and running *p* >0.05 but statistically non-significant. In contrast, girls significantly dominate over boys in balancing t(466) = −2.19, *p* < .05 and jumping t(466) = −6.07, *p* < .01 (Table 2).

**Table 2:** Differences in individual basic motor competencies according grade and gender.

Test items	3rd grade		4th grade		boys		girls		total	
	M	SD	M	SD	M	SD	M	SD	M	SD
Object movement										
Throwing	0.68	0.71	0.75	0.73	0.81	0.74**	0.61	0.68	0.71	0.72
Catching	0.95	0.80	1.09	0.76	1.17	0.77**	0.86	0.76	1.02	0.78
Bouncing	1.16	0.82	1.23	0.78	1.36	0.76**	1.02	0.81	1.19	0.80
Dribbling	1.29	0.76	1.40	0.69	1.58	0.60**	1.10	0.76	1.35	0.72
Self-movement										
Balancing	1.37	0.73	1.50	0.67*	1.36	0.71	1.50	0.69*	1.43	0.70
Rolling	1.09	0.89	1.34	0.86**	1.27	0.87	1.17	0.91	1.22	0.89
Jumping	0.39	0.69	0.59	0.77**	0.30	0.62	0.70	0.80**	0.49	0.74
Running	1.40	0.76	1.64	0.58**	1.57	0.67	1.48	0.70	1.52	0.68

* = *p* < 0.05.

** = *p* < 0.01.

**Figure 1:**
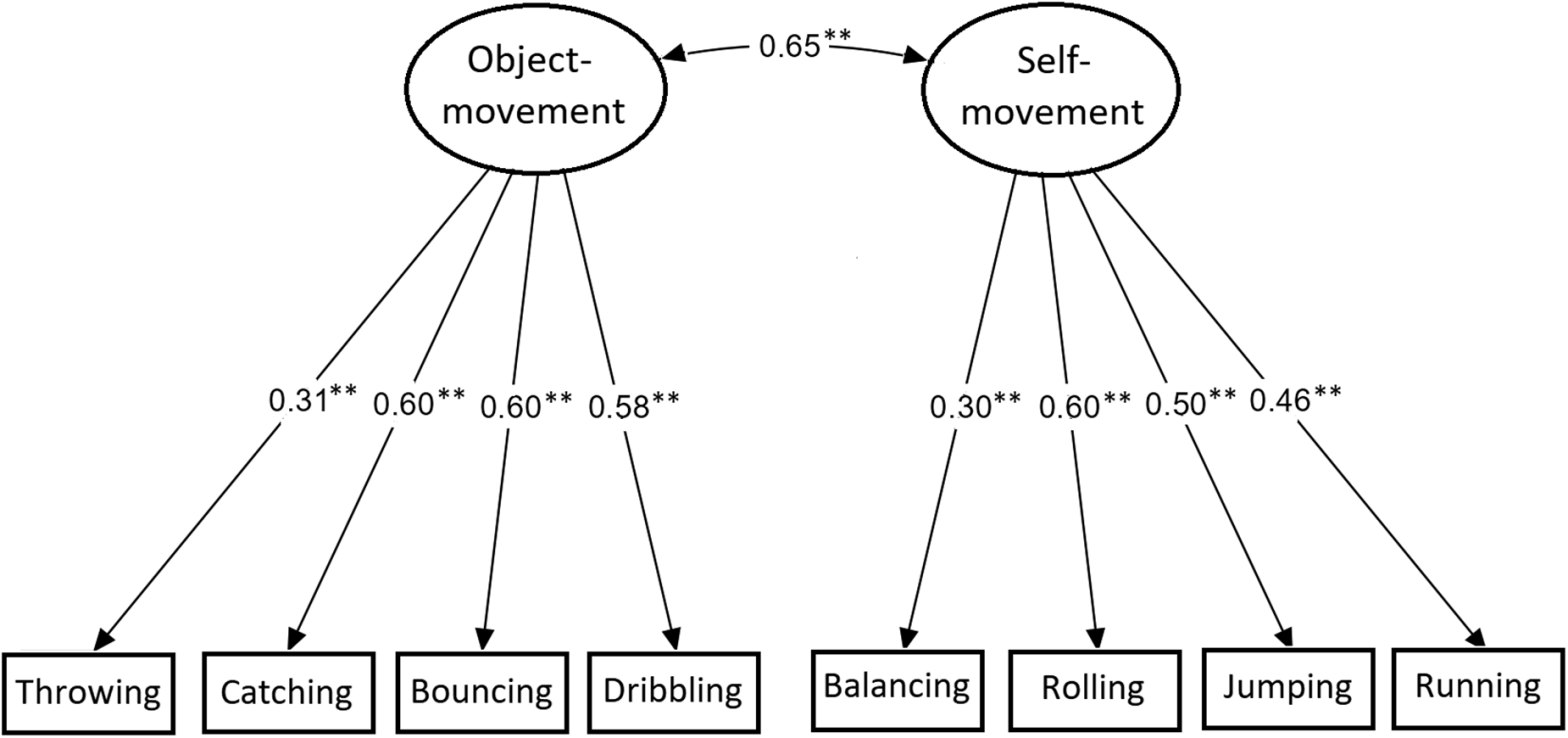
Confirmatory factor analysis.

In our confirmatory analysis to test construct validity, we confirmed the assumed theoretical two-factor structure of the MOBAK 3–4 test items in our sample from different part of Slovak Republic. Specifically, in the Model 1 test, we showed a sufficient model fit *χ*2 = 35.64; *df *= 19; *p* < .05; confirmatory fit index = 0.96; root mean square error of approximation = 0.043) with factor loadings ranging between.30 and.60 and a correlation between the two factors of r = 0.65 (Figure 1).

We hold that the changes in boys between the 3rd and 4th grade elementary school have a greater positive effect on their potential to perform object movement tasks compared to the girls. However, the situation is quite the opposite in self-movement activities. The 3rd grade boys appear to do better in object movement only t(230) = 5.93, *p* < .01 compared to the girls of the same age. The performance in the self-movement activities of the 3rd-grade boys and girls varies *p* >0.05. In the 4th grade, the situation is generally different. The boys are better at object movement t(234) = 4.59, *p* < .01 and, on the other hand, the girls are better at self-movement t(234) = −3.64, *p* < .01. The results of the comparison in the detected level of basic motor competencies in the 3rd-graders and 4th-graders together by gender show a gender conditionality for a successful performance of motor tasks in the basic motor competencies test items, in Object-movement boys achieved better results t(466) = 7.47, *p* < .01 and girls appear to do better in self-movement t(466) = −1.97, *p* < .05.

One possible assessment of the potential differences in basic motor competencies in 3rd-graders and 4th-graders is to compare their performance without distinguishing their gender. It turns out that in both basic motor competencies, i.e., object-movement t(466) = −1.99, *p* < .05 and self-movement t(466) = −4.60, *p* < .01, the 4th-graders had better results than their 3rd grader peers. The comparison of the performance of boys and girls in relation to the class they attend, and thus their age being an accompanying attribute in the transition from the 3rd to the 4th grade, points to some differences. The development changes in the 4th-graders boys compared to 3rd-graders boys did not produce a statistically validated improvement in performance neither in object movement nor in self-movement activities *p* > 0.05. On the contrary, the 4th-grade girls achieved a higher level of basic motor competencies than the 3rd-grade girls both in object movement t(226) = −2.14, *p* < .05 and self- movement t(226) = −5.21, *p* < .01 activities (Table 3).

**Table 3:** Differences in object-movement and self-movement according grade and gender.

	Object movement				Self-movement			
	boys		girls		boys		girls	
	M	SD	M	SD	M	SD	M	SD
3rd grade	4.84	1.93**	3.33	1.97	4.32	1.86	4.19	2.01
4th grade	5.00	1.96**	3.87	1.82	4.65	1.82	5.53	1.86**
total	4.93	1.94**	3.59	1.92	4.49	1.85	4.85	2.05*
	3rd grade		4th grade		3rd grade		4th grade	
	M	SD	M	SD	M	SD	M	SD
boys	4.84	1.93	5.00	1.96	4.32	1.86	4.65	1.82
girls	3.33	1.97	3.87	1.82*	4.19	2.01	5.53	1.86**
total	4.09	2.09	4.46	1.98*	4.25	1.94	5.07	1.89**

* = *p* < 0.05.

** = *p* < 0.01.

**Figure 2:**
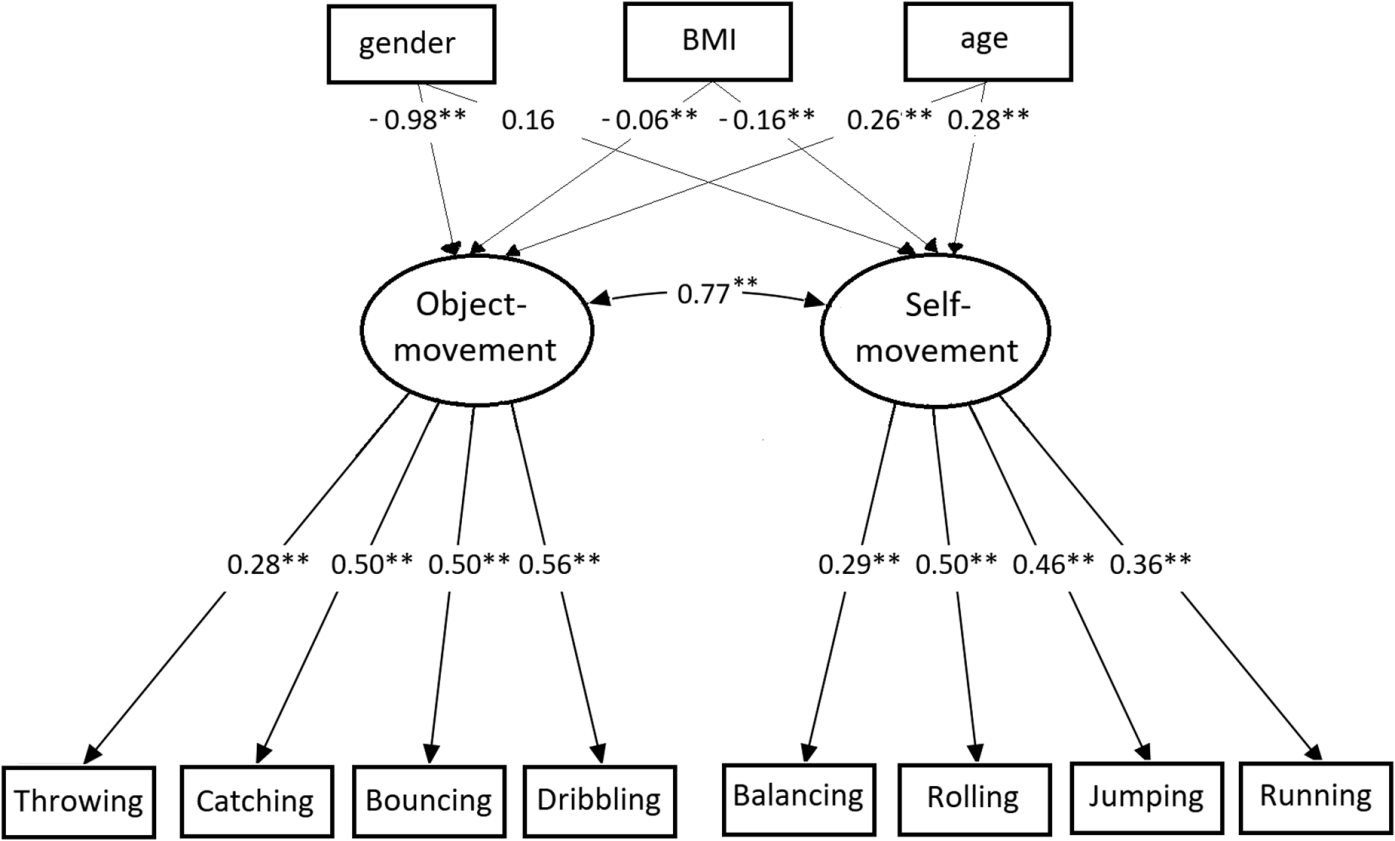
Confirmatory factor analysis with the covariates gender, BMI, and age.

For the factorial validity of Model 2, which considers the gender, BMI, and age as covariates, the results of the indices of fit are: *χ*2 = 127.63; df = 45; *p* < .01; CFI = 0.87; RMSEA = 0.063. Both the CFI index and RMSEA are slightly below the recommended level of fit. Gender has a strong relationship with object movement and a small non-significant relationship with self-movement. Boys have higher values than girls for object movement, while girls have higher values for self-movement. BMI has a small negative but significant relationship with object movement and self-movement too. Children with a low BMI had higher values. Age also displays a moderate relationship with object movement and self-movement where older children display higher values (Figure 2).

## 4 Discussion

Knowing the level of BMC is a prerequisite for improving the quality of the educational process in physical education. For this reason, a reliable tool for their identification is also needed. The main goal was to contribute to the knowledge of BMC in the 3rd and 4th grade boys and girls from elementary schools in the Slovak Republic and to determine the construct validity of the MOBAK battery also with the covariates gender, BMI and age.

In the previous research report (24), we found that the 1st-grade and 2nd-grade boys showed a higher performance than girls in object movement activities (competency) when comparing the performance of 1st and 2nd grade boys and girls. The situation in object movement activities is identical even in the case of 3rd grade and 4th grade boys who are better at object movement activities than their female peers. The results so far indicate that the Slovak boys are better at object movement activities than the girls from the 1st to 4th grade.

The comparison of 3rd and 4th grade girls with the boys in self-movement activities does not replicate the situation when comparing the 1st and 2nd grade girls with their male peers where the girls scored better than the boys (24). The 3rd grade girls had a lower average performance in self-movement activities than the 3rd grade boys, although not statistically significantly.

The 1st to 4th grade girls showed a better trending in the self-movement activities compared to their male peers except for the 3rd graders.

Slovak third and fourth graders of both genders achieved a higher performance level compared to selected German peers (10). The comparison with pupils from Chile (2) and Athens, Foggia and Lisbon (4) is again in favor of the examined group of Slovak children. Pupils in the cities of Salzburg, Kaunas, and Zurich achieved a higher level of BMC, while a similar level to examined Slovak children was recorded in the cities of Brno and Gronigen (4). The comparison of two regions of Slovakia is interesting: the children of central and northern Slovakia we studied reached a lower level in object movement and a higher level in self-movement than their peers from the City of Trnava (4).

The factors influencing the level of BMC in object movement and self-movement of the 3rd- and 4th-graders also include age. The low impact of age on the level of BMC is generally noted by Scheuer, Bund & Herrmann (10, 24). The effect of age on both object control and self-movement activities was found (25). Other authors reported a differentiated effect of age on the level of BMC. Carcamo, Oyarzun and Herrmann (2) note the impact of age on the level of object control activities and a slight effect on self-movement activities, while Strotmeyer, Kehne and Herrmann (26) have not confirmed the impact of age on self-movement activities. The results are not unambiguous and need to be differentiated according to the impact of age on object movement or self-movement.

Are there any differences in the BMC according to gender in the 3rd- and 4th-graders? It seems that the results of past research by various researchers are proving to be clearer in this respect. The boys score higher in object movement activities than the girls (2, 24–27). Our results are consistent with the above. In the self-movement activities, we did not find any differences between the boys and girls, which is consistent with the previous results (25, 27). On the contrary, other studies found the girls to perform better in self-movement activities than the boys, and/or reported a slightly better performance in girls (2, 17). The above suggests that to answer the question about the potential impact of gender on the BMC of 3rd- and 4th-graders, it will be necessary to make a constant distinction between object control activities and self-movement activities, and decide whether the examination concerns the 3rd and 4th grades separately with an age dispersion of 12 months, or two grades combined, i.e., 3rd-graders and 4th-graders together in one test group with an age dispersion of approximately 24 month. It has been shown that the gender-related differences increase along age cohorts. Such differences are explained to be due to different opportunities for motor experiences (1).

If we focus on confirmatory analysis, our model achieved satisfactory fit index values according to the recommendation (23). The correlation between the object-movement and self-movement parameters is similar to that in research (2, 10, 12), but the factor loadings are slightly lower. Gender, BMI and age appeared as significant covariances (2, 4) in our research as well. Gender determined the object movement parameter to almost 100%, and BMI, even with a very low coefficient, appeared as a significant covariance. A more detailed view of psychomotor development and the level of motor competences is provided by the research of Martinez (28), and subsequent findings confirm that overweight represented by a higher BMI value affects children's ability to perform tasks related to object control and self movement (29).

The interesting finding by Herrmann and Seelig (10) that “*competency profiles of these classes are connected to the external criteria sex, type of sport, and amount of physical activity, what indicates that the type and amount of physical activity can be represented in the basic motor competencies”* should be verified in the Slovak environment in further research. These claims are also supported by Madrona's research (30) where the results show that those children who performed extracurricular activities related to physical activities and sports had a greater development of laterality and postural tonic control than those who did not attend this type of activities. It will be necessary to theoretically update the rationale behind the need for national comparisons (31, 32). Does it mean that the children in one country are better at BMC than those from another country? Does a particular country have a better state-run education system, or do children spend more time pursuing organized or casual physical activities? We could readily formulate other similar scientific and speculative questions.

Together with the study (4), almost all of Slovakia is covered and we can state that the results of this research and the resulting findings are relatively valid in the educational environment of the Slovak Republic, and they relate to children of the 3rd and 4th grade in junior elementary schools in the observed age range.

In terms of age, the division of children by school “class” should be deprecated in future research and a stricter age-based division should be implemented even at the cost of some children's primary data being moved to the primary data of children in the lower or upper grade. However, we are aware that this would result in a mix of children with different educational experience, as the individual groups would include the children with different length of organized education, and with the focus on physical education or other subjects.

Another option would be to adhere to the original division by “class” but exclude those children from the analysis of primary data whose age is lower or higher than the vast majority of other children in the class. Unfortunately, this would result in an overall decrease in the number of subjects in the individual groups, however, these children would have a more comparable organized and casual educational background.

These methods would result in a greater age homogeneity of the compared groups. Ideally, a uniform procedure in all research dedicated to children's basic motor competencies should be selected and introduced. This would further increase the objectivity of comparisons.

The fundamental methodological question is to justify the need to compare the basic motor competencies in children from different countries. Our speculative explanation of this phenomenon is that the higher level of basic motor competencies in the children from a particular country may be determined by a better educational process in the organized, casual and other activities in these countries. The achievements in these countries could become a model for other countries with lower levels of basic motor competencies in children.

## 5 Practical implications

A consistent implementation of the fundamental motor skills and abilities in the educational environment in the 3rd and 4th grade elementary school in the Slovak environment requires a manual in the Slovak language for the teachers, which will be written with the educational terminology used in the Slovak elementary schools (33, 34).

## 6 Conclusions

We have identified the following trends: (i) the boys had higher performance levels in object movement activities than the girls. However, the situation in self-movement activities was not as clear in favor of the girls as it was in favor of the boys in the case of object movement activities. Only the 4th-grade girls showed better results than the boys. The general expectation that the age difference after the transition from the 3rd to 4th grade is a general factor with an effect on the improvement of performance in BMC, was not clearly verified. This assumption was only confirmed in girls.

Generally, in terms of the impact of age on the performance in BMC, the age range of the studied groups should always be taken into account. The following logical expectation may apply: the smaller the age range of the studied group, the smaller the effect of age on the performance in BMC, and vice versa.

## Data Availability

The raw data supporting the conclusions of this article will be made available by the authors, without undue reservation.

## References

[1] Herrmann C Heim C Seelig H. Construct and correlates of basic motor competencies in primary school-aged children. J Sport Health Sci. (2019) 8:63–70. 10.1016/j.jshs.2017.04.002 30719385 PMC6349583

[2] Carcamo-Oyarzun J Herrmann C. Validez de constructo de la batería MOBAK para la evaluación de las competencias motorices básicas en escolares de educatión primaria. (construct validity of the MOBAK test battery for the assessment of basic motor competencies in primary school children). Rev Española de Pedagog. (2020) 276:291–308.

[3] Lopes L Santos R Coelho-e-Silva M Draper C Mota J Jidovtseff B A narrative review of motor competence in children and adolescents: what we know and what we need to find out. Int J Environ Res Public Health. (2021) 18:18. 10.3390/ijerph18010018 PMC7792958 33375134

[4] Wälti M Seelig H Adamakis M Colella D Emeljanovas A Gerlach E Investigating levels and determinants of primary school children’s basic motor competencies in nine European countries. Zeitschrift für Grundschulforschung. (2022) 16:113–33. 10.1007/s42278-022-00155-w

[5] Ludyga S Mücke M Kamijo K Andrä C Pühse U Gerber M The role of motor competences in predicting working memory maintenance and preparatory processing. Child Dev. (2019) 91(1):799–813. 10.1111/cdev.13227 30791099

[6] Van der Fels IMJ Te Wierike SCM Hartman E Elferink-Gemser MT Smith J Visscher C. The relationship between motor skills and cognitive skills in 4−16 year old typically developing children: a systematic review. J Sports Sci Med. (2015) 18(6):697–703. 10.1016/j.jsams.2014.09.007 25311901

[7] Schierz M Thiele J. Weiter denkenUmdenken-neu denken? In: Aschebrock H Stibbe G, editors. Didaktische Konzepte Für Den Schulsport.; Aachem. Meyer & Meyer (2013). p. 122–47.

[8] Estevan I Barnett LM. Considerations related to the definition, measurement and analysis of perceived motor competence. Sports Med. (2018) 48(12):2685–94. 10.1007/s40279-018-0940-2 29938348

[9] Leonard HC. The impact of poor motor skills on perceptual, social and cognitive development: the case of developmental coordination disorder. Front Psychol. (2016) 7:1–4. 10.3389/fpsyg.2016.00311 27014127 PMC4779971

[10] Herrmann C Seelig H. Structure and profiles of basic motor competencies in the third grade—validation of the test instrument MOBAK-3. Percept Mot Skills. (2017) 124(1):5–20. 10.1177/0031512516679060 27879423

[11] Allal L. Assessment and the regulation of learning. In: Peterson B Baker E McGaw B, editors. International Encyclopedia of Education. Vol. 3. 2nd ed. USA: Elsevier (2010). p. 348–52.

[12] Quitério A Martins J Onofre M Costa J Mota Rodrigues J Gerlac E Mobak 1 assessment in primary physical education: exploring basic motor competences of Portuguese 6-year-olds. Percept Mot Skills. (2018) 125(6):1055–69. 10.1177/0031512518804358 30413140

[13] Herrmann C Gerlach E Seelig H. Development and validation of a test instrument for the assessment of basic motor competencies in primary school. Meas Phys Educ Exerc Sci. (2015) 19(2):80–90. 10.1080/1091367X.2014.998821

[14] Hulteen RM True L Pfeiffer KA. Differences in associations of product- and process-oriented motor competence assessments with physical activity in children. J Sports Sci. (2019) 4:375–82. 10.1080/02640414.2019.1702279 31847740

[15] Herrmann C Seelig H. Construct validity of the MOBAK-5 test instrument and determinants of basic motor competencies of fifth graders. Ger J Exerc Sport Res. (2017) 2:110–21. 10.1007/s12662-016-0430-3

[16] Herrmann C Ferrari I Wälti M Wacker S Kühnis J. MOBAK—kG. Basic Motor Competencies in Kindergarten. Testmanual. 2nd ed. Basel: University of Basel, Luxembourg (2018). p. 28. Available at: http://mobak.info/en/wp-content/uploads/2018/11/MOBAK-KG_engl.pdf (Accessed on January 19, 2023).

[17] Herrmann C Seelig H Ferrari I Kühnis J. Basic motor competencies of preschoolers: construct, assessment and determinants. Ger J Exerc Sport Res. (2019) 2:179–87. 10.1007/s12662-019-00566-5

[18] EUROFIT. Handbook for the EUROFIT Tests of Physical Fitness. 2nd ed. Strasbourg: Publishing and Documentation Service Strasbourg, Sports Division Council of Europe (1993). p. 75.

[19] Herrmann C Seelig H. MOBAK—3. Basic Motor Competencies in Third Grade. Testmanual. University of Basel: Basel (2015). p. 24. Available at: http://www.dsbg4public.ch/custom/upload/docs/i7byrjbq3ms4qczh9b2bdkvqsx1zio21253g.pdf (Accessed on January 19, 2023)

[20] Herrmann C Seelig H Ch H Kehne M Gerlach E. MOBAK 1-4. Test Zur Erfassung Motorischer Basiskompetenzen Für Die Klassen 1-4. 1st ed. Göttingen, Germany: Hogrefe Verlag GmbH & Co. KG (2018). p. 75.

[21] Mačura P Blahutová A Hubinák A Testové batérie MOBAK. [MOBAK test batteries]. (in Slovak). Šport Eduk. (2018) 2:12–58.

[22] Scheuer C Bund A Herrmann C. Diagnosis and monitoring of basic motor competencies among third-graders in Luxembourg. An assessment tool for teachers. Meas Phys Educ Exer Sci. (2019) 3:258–71. 10.1080/1091367X.2019.1613998

[23] Hu L Bentler PM. Cut-off criteria for fit indexes in covariance structure analysis: conventional criteria versus new alternatives. Struct Equ Modeling. (1999) 6(1):1–55. 10.1080/10705519909540118

[24] Mačura P Blahutová A Hubinák A Basic motor competencies in the 1st and 2nd grade elementary school children in Slovakia. In: Cacek J Sajdlová Z Šimková K, editors. Proceedings of the 12th International Conference on Kinanthropology. Sport and Quality of Life, Brno, Czech Republic, November 7-9, 2019. Brno, Czech Republic: Masaryk University Press (2020). p. 74–83.

[25] Kossyva I Manolis A Herrmann C. Evaluation of Basic Motor Competencies in Primary School Children: Validity of MOBAK-3 Test Instrument in Greece. Verona, Italy: Event: Healthy and Active Children (2019).

[26] Strotmeyer A Kehne M Herrmann C. Motorische basiskompetenzen. Zusammenhänge mit geschlecht, Alter, gewichtsstatus, außerschulischer sportaktivität und koordinationsleistung. Ger J Exerc Sport Res. (2020) 1:82–91. 10.1007/s12662-019-00596-z

[27] Gerlach E Herrmann C Dania A Basic motor competencies. In: Scheuer C Bund A Holzweg M, editors. Changes in Childhood and Adolescence: Current Challenges for Physical Education. Keynotes, Invited Symposia and Selected Contribution of the FIEP European Congress, Luxembourg, Luxembourg, September 13−16, 2017. Berlin, Federal Republic of Germany: Logos Verlag Berlin GmbH (2018). p. 81–90.

[28] Martínez SJR Camacho XGO Madrona PG. Development of the checklist of psychomotor activities for 5-to 6-year-old children. Percept Mot Skills. (2018) 125(6):1070–92. 10.1177/0031512518804359 30413141

[29] Madrona PG Martinez SJR Gallego NMS Camacho XGO. Psychomotor limitations of overweight and obese five-year-old children: influence of body mass indices on motor, perceptual, and social-emotional skills. Int J Environ Res Public Health. (2019) 16(3):427. 10.3390/ijerph16030427 30717253 PMC6388181

[30] Madrona PG Martínez SJR Faraco CCR. Extracurricular physical activities and the condition of being an only child as a conditioning factor in the psychomotor development of 5-year-old children. Front Pediatr. (2021) 9:684418. 10.3389/fped.2021.684418 34291018 PMC8287093

[31] Vrbas J. MOBAK 3—presentation of a test battery of basic motor competencies and selected results of the Czech republic and Switzerland. In: Zvonař M Sajdlová Z, editors. Proceedings of the 11th International Conference on Kinantropology. Sport and Quality of Life. Brno, Czech Republic, November 29th—december 1st, 2017. Brno, Czech Republic: Faculty of Sports Studies, Masaryk University (2017). p. 125–31.

[32] Herrmann C Seelig H Wälti M Gerlach E. Assessment and Monitoring of Basic Motor Competencies in Europe. Verona, Italy: Event: Healthy and Active Children (2019). p. 18–35.

[33] Scheuer C Heck S. Modular Support-Toolkit for Teachers. Esch-Alzette: University of Luxembourg, Luxembourg (2020). p. 46.

[34] Scheuer C Heck S. Podporný Metodický Materiál Pre Učiteľov [Modular Support-Toolkit for Teachers]. Slovak, Esch-Alzette: University of Luxembourg, Luxembourg (2020). p. 44.

